# Glomerular injury after trauma, burn, and sepsis

**DOI:** 10.1007/s40620-023-01718-5

**Published:** 2023-08-05

**Authors:** Lorena Schult, Rebecca Halbgebauer, Ebru Karasu, Markus Huber-Lang

**Affiliations:** Institute of Clinical and Experimental Trauma Immunology, University Hospital Ulm, Helmholtzstr. 8/1, 89081 Ulm, Germany

**Keywords:** Glomerulus, Mesangial cell, Podocyte, Barrier, Trauma, Hemorrhagic shock, Burn, Sepsis

## Abstract

Acute kidney injury development after trauma, burn, or sepsis occurs frequently but remains a scientific and clinical challenge. Whereas the pathophysiological focus has mainly been on hemodynamics and the downstream renal tubular system, little is known about alterations upstream within the glomerulus post trauma or during sepsis. Particularly for the glomerular endothelial cells, mesangial cells, basal membrane, and podocytes, all of which form the glomerular filter, there are numerous in vitro studies on the molecular and functional consequences upon exposure of single cell types to specific damage- or microbial-associated molecular patterns. By contrast, a lack of knowledge exists in the real world regarding the orchestrated inflammatory response of the glomerulus post trauma or burn or during sepsis. Therefore, we aim to provide an overview on the glomerulus as an immune target but also as a perpetrator of the danger response to traumatic and septic conditions, and present major players involved in the context of critical illness. Finally, we highlight research gaps of this rather neglected but worthwhile area to define future molecular targets and therapeutic strategies to prevent or improve the course of AKI after trauma, burn, or sepsis.

**Graphical abstract:**

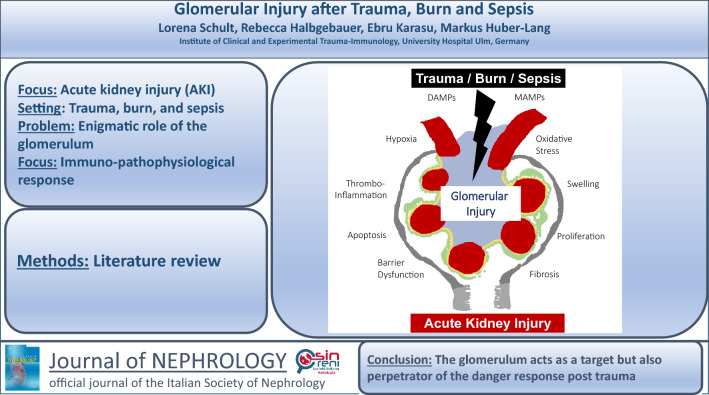

## Introduction

Following severe tissue trauma, there is an intensive immuno-pathophysiological response with multiple cross-talking systems and organs, frequently leading to complications such as multiple-organ failure syndrome and sepsis [1, 2]. External and internal barrier breakdown causes the release of a multitude of factors into the bloodstream such as cellular debris with damage-associated molecular patterns (DAMPs) and components of potentially invading pathogens (microbe-associated molecular patterns, MAMPs). These factors are recognized by fluid-phase systems such as the coagulation and complement systems as well as cellular pattern recognition receptors. Translation into an immediate and highly effective immunological response ideally results in the sealing off of injured and infected areas, opsonization of damaged tissue, clearance of tissue debris, and initiation of regenerative processes [1, 3]. However, in case of an unbalanced innate immune response, with excessive activation and subsequent dysfunction of the innate fluid and cellular “first line of defense” (represented by the complement system and neutrophils/macrophages, respectively), the initial damage with exposure to DAMPs and MAMPs may turn into systemic inflammation, loss of barrier integrity, and development of multiple organ failure [1, 2, 4]. Furthermore, excessive inflammation and/or dampened adaptive immune responses may render patients more susceptible to infectious complications [5].

The kidneys, in particular, represent a major target organ after trauma or burn or during sepsis. In this regard, the initial local and systemic pathophysiological processes caused directly by disease as well as standardized treatment strategies may cause acute kidney injury (AKI). Acute kidney injury is characterized by a multitude of alterations on a cellular and molecular level, predominantly resulting in increased local oxygen demand as well as diminished renal blood flow and thus not only a reduced glomerular filtration rate (GFR), but also impaired tubular secretion [6]. These functional shifts result in attenuated clearance and plasma accumulation of metabolites and toxins, with imminent consequences on distant organ (dys-) function [7]. According to the current state of knowledge, the major part of the complex pathophysiology of sepsis- and trauma-related acute kidney injury (TRAKI) appears to occur within the tubular system [6]. However, less is known and described in these conditions for the glomerulus. Therefore, in the present review, we focus on the significant changes within the glomerulus and its specialized cells and highlight promising future research areas and potential therapeutic strategies.

### Structure–function of the glomerular filter

The structure–function features of the glomerular filter mainly involve tight interactions between different specialized cells. The glomerular fenestrated endothelial cells are initially engaged in the filtration process. The next filtration layer is the glomerular basement membrane (GBM), a sophisticated nexus, including extracellular proteins with type IV collagen, fibronectins, proteoglycans, laminins, nidogens such as sulfated monomeric glycoproteins, and heparan sulfate proteoglycans [8]. The slit diaphragm is located in the distal filtration section, created by podocytes. Furthermore, mesangial cells, in close collaboration with podocytes, guarantee the integrity of the glomerular capillary bundle and contribute to the dynamics of the filtration process [9]. In addition to water, the filter permits small and mid-sized molecules to pass the barrier, whereas larger proteins and serum albumin are completely restricted from passing. However, all these complex structures can be damaged by different mediators generated through trauma, burn, or sepsis, which eventually can lead to functional loss. To indicate the special role of each cell type forming the glomerular filter, these are discussed in detail in the following sections focusing on traumatic, burn, and septic environments.

### Post-traumatic and septic alterations of mesangial cells

Mesangial cells reside in the center of the glomerulus and pursue multiple functions. They can regulate the tone of the vascular loops within the glomerulus, produce matrix components and inflammatory mediators, generate nitric oxide (NO), reactive oxygen species (ROS), and gasotransmitter-forming enzymes, exhibit phagocytotic activity, and can even acquire antigen-representing features [10, 11].

In the context of trauma and sepsis, in principal, mesangial cells can respond to both DAMPs and MAMPs with the generation and release of various established inflammatory mediators, including interleukin (IL)1β, Tumor necrosis factor (TNF), Monocyte Chemoattractant Protein-1 (MCP-1), and Cyclooxygenase-2 (COX-2), and thus may contribute to the inflammatory response not only locally but also systemically [12].

In a porcine model of live-bacteria-induced septic AKI, AKI development associated with enhanced renal vascular resistance was preceded by classic inflammatory mediators (IL-6 and TNF) as well as the mounting of oxidative stress [13].

In Lipopolysaccharide (LPS)-induced AKI in pigs, histological analyses revealed enhanced staining for IL-1β and TNF in mesangial cells [14]. Moreover, in pigs with *Escherichia-coli*-neurotoxin-induced septic shock, morphological investigation of the kidneys revealed an overall mesangial widening and an increase of the mesangial volume and nuclei numbers, as well as an enlarged surrounding matrix. These alterations were accompanied by a quantitative increase of ribosomes, rough endoplasmatic reticulum, and lysosomes [15].

**Fig. 1:**
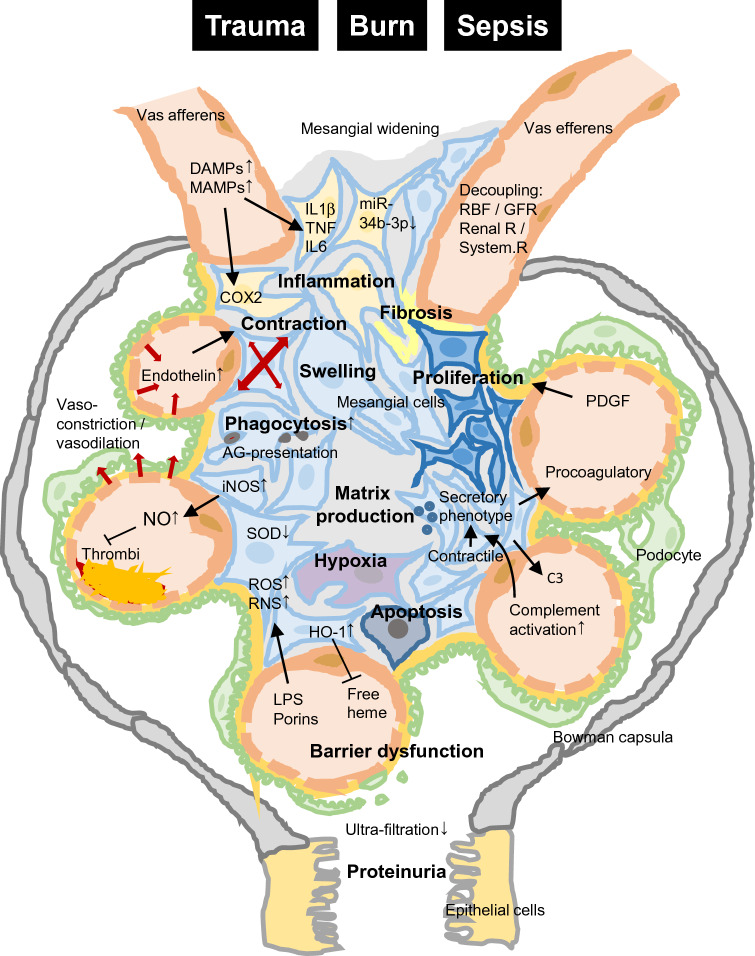
Changes in the glomerular structure after trauma, burn, and sepsis. Inflammation triggered by DAMPs and MAMPs, hypoxia, cell swelling, proliferation, contraction, and fibrosis as well as an increase of phagocytosis and matrix production lead to barrier dysfunction. Proteinuria as a sign of glomerular injury can be detected. *AG* antigen, *COX* cyclooxygenase, *DAMP* damage–associated molecular patterns, *GFR* glomerular filtration rate, *HO-1* heme oxygenase, *IL* interleukin, *iNOS* inducible nitric oxide synthase, *LPS* lipopolysacharide, *MAMP* microbe-associated molecular patterns, *NO* nitric oxide, *PDGF* platelet-derived growth factor, *RBF* renal blood flow, *RNS* reactive nitrogen species, *ROS* reactive oxygen species, *SOD* superoxide dismutase, *TNF* tumor necrosis factor

Regarding blood pressure alterations during homeostasis and in pathological conditions, mesangial cells with their contractile fibers can regulate glomerular flow via the generation of NO. Components of the renin–angiotensin–aldosterone-system (RAAS) also differentially contribute to systemic blood pressure regulation. Injection of live gram-positive or -negative bacteria in rats led to NO synthase (isoform II) upregulation in the kidneys, resulting in a corresponding vasodilation and hypotension [16]. In the same study, in vitro exposure of mesangial cells to NO and inflammatory mediators (IL1β and TNF) synergistically downregulated the angiotensin II type 1 receptor I, which in principle could induce hypotensive reactions during sepsis [16]. In agreement with this, in vitro [17] or in vivo LPS administration in rodents—in an attempt to simulate some features of septic conditions—resulted in enhanced NO synthase immunoreactivity not only in macrophages but also in mesangial cells, and, furthermore, in downregulation of mesangial Cu/Zn superoxide dismutase (SOD) [18, 19]. Enhanced NO formation facilitates the generation of the highly toxic peroxynitrite (NO plus superoxide can form ONOO^−^). Because the dismutation of superoxide appears to be impeded (by the reduced SOD), this in turn can lead to enhanced radical stress in the glomerulus. In support of this, LPS or TNF stimulation of mesangial cells resulted in cyclic guanosine monophosphate upregulation and L-arginine-derived NO and, thereby, could add to glomerular capillary vasodilation [20]. Of note, NO generation by mesangial cells not only altered regional blood flow but also greatly prevented the formation of LPS-induced glomerular thrombi in the in vivo setting [21]. In this context, it is tempting to speculate that platelet activating factor which drives platelet aggregation, and which is generated by human mesangial cells upon exposure to MAMPs (e.g., to porins or LPS), acts as a contributing mechanism to sepsis-induced thromboinflammation and thrombus formation within the glomerular loops [22], as shown in Fig. 1. Furthermore, mesangial cells release tissue plasminogen activator and an excess of plasmin activator inhibitor-1, the latter of which has been suggested to protect the basal membrane from proteolytic attack [23].

However, upon LPS stimulation, this fibrinolytic balance of mesangial cells could switch to tissue factor-like pro-coagulatory activity [24]. Thrombi in the glomerular loops are also frequently present in the case of crush-injury, and clinically evident in major shock with myoglobinuria and signs of AKI [25]. However, the underlying mechanisms and possible involvement of mesangial cells for the development and resolution of these thrombi remain unclear.

In regard to the renin angiotensin aldosterone system (RAAS) axis, in vitro exposure of mesangial cells to LPS resulted in significantly reduced angiotensin I and II generation and release [26, 27]. The resulting vasodilatory effects could in principle counteract the proposed uncoupling of the systemic and renal blood flow as well as the uncoupling of the renal blood flow from the GFR during sepsis [13, 28]. By contrast, significantly enhanced endothelin concentrations—which can originate from endothelial cells but also from mesangial cells [29]—have been detected in the circulation within 48 h after burn injury (> 20% of the body surface area). In turn, endothelin may lead to renal mesangial vessel contraction [30]. In addition, the acute phase protein C-reactive protein, which is significantly enhanced early after burn injury but also during sepsis and after trauma, is not only capable of inhibiting NO generation but also of enhancing ROS release by mesangial cells [31].

During sepsis, increased levels of the long non-coding RNA nuclear enriched abundant transcript-1 (NEAT1) could be detected in blood, which correlated with the degree of sepsis-induced AKI. Furthermore, in vitro, NEAT1 exacerbated LPS-induced mesangial cell injury in a nuclear factor k-light-chain enhancer of activated B cell- (NFkB) dependent manner [32]. In rodent sepsis induced by cecal-ligation-and-puncture (CLP), clinical signs of AKI, including enhanced retention parameters and renal damage markers, were detected together with a massive reduction in renal blood flow. This functional impairment was associated with some glomerular enlargement. These changes were reversed by treatment with the polyphenol compound resveratrol, which exhibits anti-inflammatory and anti-oxidative features [33].

Although it is established that LPS-exposure models poorly reflect the complex immuno-pathophysiology of human sepsis [34], they may help in defining basic mechanisms in mesangial cells. Numerous in vitro studies indicated that LPS exposure of mesangial cells results in multiple expression, signaling, and functional changes [35]. For example, adding LPS to mesangial cells led to enhanced TNF, IL-1β and IL-6 production and reduced miR-34b-3p generation [35]. These results were also found in a murine model of septic AKI (induced by CLP), where enhanced serum creatinine, blood urea nitrogen, TNF, IL-1β and IL-6 levels were detected and were associated with decreased kidney expression of miR-34b-3p and histological signs of reduced tissue damage [35]. Of note, in vivo administration of miR-34b-3p agomir improved septic AKI development.

In translational studies, proteome profiling of renal live biopsies during normodynamic porcine sepsis (induced by live *Pseudomonas aeruginosa* infusion, which was associated with a > 40% decrease in GFR) revealed a time-dependent alteration of approximately 20 proteins. These altered proteins were mainly attributed to cellular distress (including free radical scavengers) and repair mechanisms [36]. However, these findings refer to the entire kidney and cannot account for the glomerular response alone.

Cellular hypoxia after severe trauma or burn injury and during sepsis results in the generation of erythropoetin. This glycoprotein, which is a member of the cytokine superfamily, is mainly released by the kidneys. Erythropoetin administration revealed differential effects: while insignificant changes were reported in an endotoxin-induced sepsis model, it clearly improved renal function in rodents after trauma-induced hemorrhagic shock. However, although mesangial cells do express the erythropoetin receptor, its role after trauma/burn/sepsis remains to be defined [37].

In polytraumatized patients who developed septic complications, a proteomic approach revealed that the cytoprotective heme-oxygenase-1 (HO-1), which is crucially involved in free heme degradation, was upregulated in blood leukocytes [38]. Heme is considered to be a powerful DAMP [39]. In accordance, the role of free heme and HO-1 could also be of interest in glomerular pathophysiology. In rat mesangial cells, Lipopolysaccharide challenge did indeed upregulate HO-1, indicating some protective cellular effects against oxidative stress [40]. Further evidence in CLP-induced sepsis corroborated that HO-1/miR-218-5p signaling is important for septic AKI development and confirmed LPS-induced dysfunction of glomerular mesangial cells [41]. Whether HO-1 application will help to prevent TRAKI [6] needs further basic investigation and eventually, clinical translation.

Trauma, burn, and sepsis are associated with a robust local and systemic activation of the complement system with the generation of the anaphylatoxins C3a and C5a. In principle, these complement activation products can induce all classical signs of inflammation and barrier dysfunction [27, 42, 43]. Of note, C3a stimulation of mesangial cells altered the contractile phenotype to a secretory function [44]. Furthermore, mesangial cell exposure to the terminal complement complex (TCC, sC5b–9) resulted in the generation of prostaglandins and auto-growth factors (Mast cell interleukin 1). Furthermore, TCC also led to an alteration of the fatty acid features of the membrane phospholipids of mesangial cells [45]. However, whether and to what extent these will result in long-term effects after traumatic or septic conditions remains to be determined. Moreover, mesangial cells can locally produce the central complement component C3 [44]. Complement activation can also result in deposition of C3 cleavage products, such as C3b on the membrane surface, which are crucial for opsonization of mesangial cells and potential, subsequent phagocytotic clearance. However, whereas in the development of glomerulonephritis, C3 deposition can be a crucial part of the pathophysiological process [46], in the case of trauma and sepsis, this does not appear to be a leading mechanism. Additionally, debris clearance of traumatized or infected cells involves phagocytotic processes, which can also be accomplished by mesangial cells themselves [11].

In addition to the fluid phase innate immune response, apoptotic processes are important to clear injured cells and debris (including damaged mesangial cells) before repair mechanisms can occur. Lipopolysaccharide exposure of mesangial cells can induce apoptotic events [32]. Moreover, a fine-tuned balance between ROS and reactive nitrogen species has been proposed for the induction of mesangial apoptosis [47]. Following debris clearance, repair and proliferation processes may in principle involve mesangial cells, also in the trauma or sepsis setting. Platelet-derived growth factor (PDGF)-B plays a crucial role in mesangial cell proliferation [48]. Elevated serum PDGF-B concentrations were indeed detected after trauma or during sepsis [49, 50]. Other trauma- or sepsis-relevant factors may also increase mesangial cell proliferation and may even support fibrotic processes. In vitro exposure of mesangial cells to either oligonucleosomes or High-mobility-group-protein B1 (HMGB-1), both of which are massively generated after polytrauma [1, 51], resulted in mesangial cell proliferation and matrix generation [52–54]. However, sustained in vivo mesangial proliferation after exposure to DAMPs and MAMPs in traumatic, burn, and septic environments remains to be proven.

Overall, it seems likely, but still needs clinical clarification, that mesangial cells contribute to renal damage in response to DAMPs and MAMPs and circulatory shock conditions by local generation of inflammatory mediators such as TNF [14] and matrix components, vaso-regulation, cytoplasmic enlargement, and proliferation [55]. In the clinical setting of severe burn injury, mesangial widening, cell proliferation and hypertrophy, and occlusion of the associated capillary loops were described after lethal outcome even in the absence of evident clinical AKI signs, indicating development of burn-induced glomerulopathy [56].

### Functional shift of glomerular endothelial cells after trauma, burn, or sepsis

The kidney possesses one of the richest and most heterogeneous endothelial cell populations, including those found in the glomerular endothelium and microvascular endothelium in peritubular capillaries [57]. These cells display unique characteristics because they need to maintain homeostasis in the presence of environmental extremes in terms of both oxygenation and osmolality [58].

Comparable to other endothelial cells, renal endothelial cells form the interface between the blood and tissue compartments, regulate the vasomotor tone, and contribute to perivascular tissue hemostasis. In addition, these cells are crucially involved in immune cell trafficking. Of note, glomerular endothelial cells (GECs) differ remarkably from most other endothelial cells because they are extraordinarily flattened and highly fenestrated. In this differentiated form, they enable glomerular ultrafiltrate generation [59]. Although significant progress has been achieved in the research of endothelial biology, little is known about the different renal endothelial populations and the involved molecular mechanism after trauma and sepsis driving kidney endotheliopathy. This lack of knowledge is possibly due to the complex and multi-factorial pathophysiology of kidney endotheliopathy during AKI, which includes intra-renal hemodynamic instabilities due to posttraumatic shock, glycocalyx and endothelial dysfunction (including glycocalyx shedding), and infiltration of inflammatory cells, as well as exposure to debris and pathogens [6].

Nevertheless, the renal vascular endothelium is known to be a primary target in several disease processes, including ischemic acute renal failure, trauma, and sepsis. The post-traumatic inflammatory response releases multiple danger molecules, including DAMPs and MAMPs, histones, free heme, DNA, RNA, and HMGB-1, among others [1]. During inflammatory states, including trauma and sepsis, ECs come into contact with these danger molecules and subsequent pro-inflammatory mediators that profoundly change their physiologic functions with a shift from an anti-inflammatory to a pro-inflammatory phenotype [60]. Consequently, interaction with and adhesion of leukocytes to ECs is facilitated, and ECs also switch from an anti-coagulatory state to a pro-coagulatory state. All of this can result in an altered EC barrier function, leading to increased permeability and impaired vasomotor tone [58].

Glomerular endothelial cells closely interact with podocytes and mesangial cells in a triple directional crosstalk. Therefore, GEC dysfunction initiates a molecular shift from a “victim” to “driver” of kidney injury, reflected by podocyte damage, proteinuria, and mesangial activation [61]. Furthermore, shedding of the endothelial barrier molecules has been reported in both sepsis and trauma patients as a key contributor to barrier disruption as well as organ dysfunction [61, 62]. Of note, an additional hemorrhagic shock in trauma patients known to cause various problems to the endothelium is correlated with the clinical severity of organ failure [62]. Another study reported that shedding of the vascular endothelial cadherin is associated with the severity of acute kidney injury and multiple organ dysfunction in patients suffering from sepsis [63]. Damage to the endothelial glycocalyx is also associated with abnormal vascular permeability. In this context, studies revealed a crucial role of TNF in the development of renal endothelial dysfunction. In vitro studies on primary cultures of mouse and human renal endothelial cells suggest that TNF exposure increases the permeability to albumin [64]. In vivo, LPS-induced acute endotoxinemia caused impairment of the glomerular endothelium by a decreased abundance of heparan sulfate proteoglycans and sialic acid. These changes were associated with the manifestation of albuminuria [65]. Lipopolysaccharide exposure also decreased the GFR and caused structural alterations in the glomerular endothelium. Significant changes in the density and diameter of glomerular endothelial cell fenestrae were observed in LPS-challenged mice compared to control littermates [65]. Mechanistically, TNF interaction with the TNF receptor-1 (TNFR-1) was found to be critically involved in the pathophysiology of the kidney endothelium. In TNFR-1-knockout mice, the effects of LPS on the glomerular endothelial surface layer, endothelial cell fenestrae, GFR, and albuminuria were all diminished. By contrast, intravenous TNF administration further decreased the GFR and led to a loss of glomerular endothelial cell fenestrae, increased fenestrae diameter, and damage to the glomerular endothelial surface layer [65].

Although sepsis is frequently associated with systemic vasodilation and can occur in the context of high, normal, or low cardiac output, an increase in renal vascular resistance (RVR) appears to be a key hemodynamic factor associated with sepsis-induced AKI mainly independently of the renal blood flow [66]. In post-cardiac surgery patients with a defined “surgical tissue trauma”, a significant increase in RVR and decrease in renal blood flow was considered indicative of microvascular alterations, including vasoconstriction and likely capillary leak with subsequent tissue edema [67].

Other studies proposed that pericytes are important for endothelial stabilization and are critically involved in the pathophysiological process of AKI. Upon developing sepsis, pericytes are activated, can detach from the endothelium of peritubular capillaries [68], and migrate to the interstitium, where they can differentiate into myofibroblasts [69]. Therefore, pericyte detachment from the endothelium promotes an unstable and leaky endothelium, which contributes to the inflammatory cascade and oxidative stress [70]. In turn, oxidative stress and ischemic conditions can induce apoptosis of the endothelial cells, further reinforcing the development of endotheliopathy in the kidneys in a “vicious circle” [70]. Here, an innovative therapy may provide a proof of concept, whereby reducing oxidative stress by mitochondria-targeted antioxidants did indeed improve the renal blood flow and permeability of the renal endothelium [71]. This concept might also represent a promising therapeutic option in hemodynamically unstable settings after severe trauma and during sepsis.

Another therapeutic approach addresses the renal metabolism: Renal endothelial cell metabolism can be altered in the context of kidney injury and diseases, partly as a result of changes in the microenvironment. For example, high levels of fatty acid oxidation (FAO) help to maintain vascular barrier integrity and provide protection against ROS [72]. Inhibition of FAO in endothelial cells increased oxidative stress, endothelial barrier permeability, leukocyte infiltration [72], and endothelial-to-mesenchymal transition [73]. Therefore, future metabolomic analysis of the kidney endothelium might also help to define sufficient therapeutic options in AKI [74], also in the context of trauma and sepsis.

### Podocytes

Within the glomerular filter, podocytes are located downstream. They can be separated into three structural and functional components: the cell body, large branching processes, and the podocyte foot. Within the Bowman´s capsule, the podocytes surround and encase the glomerular capillaries. Podocyte foot processes develop dendritic bonds with nearby located podocytes. Actin filaments within these foot processes form a contractile device which is responsible for their dynamic nature [75]. They can reorganize themselves based on the varying filtration conditions. Additionally, podocytes foster important interactions with the GBM. A series of different conjunctions are constituted by various adhesion complexes and receptors. Several receptors are responsible for guaranteeing patency of the filter barrier by interacting with actin, including integrins, syndecans, and dystroglycans. Furthermore, the GBM boundary surface involves different signaling networks of integrin and adhesion molecules. For proteins, passing this slit diaphragm represents the final step into the urinary filtrate [75].

Injured podocytes lose their structure through a process of extinction, which causes a loss of function by reducing their effectiveness as a filtration barrier. This effacement is based on the destruction of the actin cytoskeleton of podocyte foot processes [75], resulting in the appearance of larger-sized proteins in the urine.

Proteinuria is defined as an increase of proteins in the urine, while albuminuria indicates the abnormal loss of albumin. Both these clinical findings indicate a malfunction of the kidney filtration barrier [76]. In this regard, in vitro exposure of human podocyte-like epithelial cells demonstrated an albumin-induced dose-dependent increase of IL-1β, TNF, and IL-6 expression via NF-KB activation as well as the induction of apoptotic pathways with subsequent cell death [77]. Of note, caspase 3 and 7 were significantly activated as early as 2 h after albumin exposure. Overall, these findings indicate how albuminuria can increase podocyte malfunction and cell death. In turn, further exacerbation of the barrier dysfunction and progressive podocyte injury can occur, representing a “vicious circle”, particularly because podocytes have limited ability for self-regeneration and repair. Therefore, podocytes are considered a key target of kidney injury in a wide range of kidney diseases beyond trauma [78–80].

This notion is supported by studies in septic burn patients with proteinuria, suggesting a functional loss in both the tubular system and the podocyte network [81]. In cultured human podocytes, reduced expression of the slit diaphragm protein nephrin was detected after exposure to plasma from septic burn patients. These changes were accompanied by rearrangement of actin cytoskeleton fibers and the intermediate filament protein nestin as well as elevated permeability to albumin. Therefore, changes in nephrin as well as in the configuration of the cytoskeleton of podocytes might be responsible for increased permeability for albumin across the glomerular filter [81].

The severity of barrier dysfunction appears also to depend on pro-inflammatory and pro-coagulatory mediators which can be monitored in blood. In the plasma of burn-induced septic acute renal failure patients, TNF was detected, which is an established pro-apoptotic mediator [82]. Other pro-inflammatory cytokines were found in the serum of pediatric burn patients, including IL-1β, IL-5, IL-6, IL-7, IL-8, IL-13, MCP-1, and MIP-1 [83]. To what extent these mediators can cause podocyte injury remains elusive. However, it is tempting to speculate that a reduction in these inflammatory mediators and of DAMPs and MAMPs, e.g. by extracorporeal blood purification [84], which clinically seems to improve AKI in sepsis [85], might in principal help preserve podocyte function and the integrity of the glomerular filter. As proof of concept, a translational study in LPS-induced porcine sepsis and human sepsis described that enhanced CD80 expression on podocytes was responsible for increased proteinuria. Moreover, combined plasma filtration and adsorption resulted in reduced endotoxemia, reduced CD80 in the urine, and prevented proteinuria in both experimental and clinical sepsis [86].

Activated complement is an established driver of glomerular filter failure and is well described in vascular inflammation, such as antineutrophil cytoplasmic antibody- mediated renal vasculitis [87]. During sepsis, an impact of the complement activation product C5a on podocytes has been proposed, whereby it induces the secretion of pro-inflammatory cytokines like IL-6 and TNF, which can magnify tissue damage and increase ROS production. In this setting, TNF caused glomerular slit diaphragm disruption by the loss of nephrin expression and an induction of podocyte injury. Moreover, C5a was able to activate human podocytes and caused a significant decline in the cellular resistance to apoptosis [88].

In CLP-induced polymicrobial sepsis in rats, enhanced systemic C5a levels and signs of sepsis-induced AKI could be detected. Animals displayed reduced urine output, proteinuria, enhanced retention parameters, and a diminished GFR [89]. Electron microscopy of the corresponding kidneys revealed significant morphological changes in the proximal convoluted tubules 24 h after sepsis, with the loss of cell membranes, mitochondrial swelling, and intracellular edema. Furthermore, extensive foot process fusion of the podocytes was detected. Remarkably, all these functional and morphological changes could be normalized by blockade of systemic C5a [89], also improving the overall survival.

In agreement with this, another analysis of rodent kidneys after CLP-induced sepsis verified very early structural changes together with albuminuria. Systemic TNF levels in CLP-treated and sham rats increased as early as 3 h after sepsis induction. In transmission electron microscopy (TEM), some damage such as shriveled glomeruli, expanded filtration chambers, slightly expanded mesangial matrix, and microthrombi in glomerular capillaries in kidneys from septic animals was evident. Furthermore, TEM analysis of the glomeruli revealed destruction of the endothelial glycocalyx, loss of endothelial cell lining, and deprivation of podocytes. On the protein level, a significant reduction of the glycocalyx-associated proteoglycan syndecan-1 expression was detected in the kidneys harvested from CLP rats [90]. It is, therefore, tempting to speculate that regeneration of these glycocalyx components might represent a therapeutic key to maintain glomerular filter barrier function during sepsis. Albumin leakage is associated with a loss of glomerular filtration barrier selectivity as well as with significant changes in the composition of the glycocalyx [90]. It is thus conceivable that an increase in the acetylic groups of the filtration barrier-associated glycocalyx provides a compensatory mechanism, thereby preventing the desialylation of the glycocalyx, consequently limiting the activity of pro-inflammatory molecules during sepsis.

During complement cascade activation in acute inflammation, the terminal complement complex C5b–9 can affect the synthesis of collagen and profibrotic factors in human podocytes [6]. Complement activation also leads to the formation of sublytic concentrations of C5b–9 complexes on podocytes. In response, podocytes activate protein kinases, phospholipases, cyclooxygenases, and growth factors, as well as stress pathways [91]. This in turn leads to changes in the slit diaphragm, disruption of the actin skeleton, and reduced nephrin expression, eventually resulting in proteinuria [92]. Podocytes respond to surface C5b–9 complex formation with the production of mediators such as oxidants and proteases [91].

Whereas upon the insertion of high C5b–9 concentrations podocytes undergo lysis, sublytic concentrations result in a slowing-down of the cell cycle G2/M phase and in an increased synthesis of growth factors. In vitro and in vivo stimulation with sublytic C5b–9 concentrations caused DNA damage of podocytes associated with enhanced protein levels of p53, p21, Gene Growth Arrest and DNA Damage Genes, Checkpoint Kinases 1 and 2. These findings suggest that sublytic C5b–9 concentrations can cause specific DNA damage in podocytes, but do not induce apoptotic processes [93].

High p21 and p53 levels can lead to inhibition of the cyclin D/CDK4–6 complex and cause subsequent cell cycle arrest [94]. This may represent a reason for limited proliferation capabilities in podocytes after immune-mediated injury and subsequent glomerular malfunction [93]. However, it remains unclear whether and to what extent dynamically enhanced sC5b–9 concentrations, as found after severe tissue trauma [4], may alter podocyte function and morphology.

**Fig. 2:**
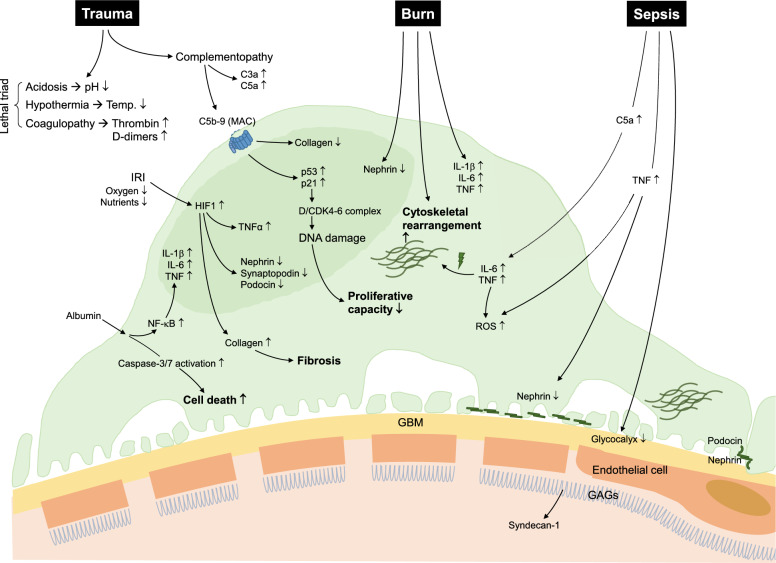
Impact of trauma, burn and sepsis on the podocyte. Cell death, fibrosis, decreased proliferation capacity, and cytoskeletal rearrangement are major effects on podocyte function. In particular, acidosis, hypothermia, and coagulopathy, known as the lethal triad, as well as IRI, complement activation, and the presence of albumin have a key role after traumatic injuries. Increased C5a and TNF in sepsis results in podocyte malfunction. Following a burn, interleukins and TNF are initiators of podocyte defect. *GAG* glycosaminoglycan, *GBM* glomerular basement membrane, *IL* interleukin, *IRI* ischemia–reperfusion Injury, *MAC* membrane attack complex, *ROS* reactive oxygen species, *Temp* temperature, *TNF* tumor necrosis factor

Irreversible damage is assumed to have occurred if inflammatory processes spread from the glomerulus to the tubulointerstitium [95]. Hypoxia in podocytes leads to enhanced intracellular concentrations of a key protein for hypoxic adaptation, hypoxia-inducible factor-1 (HIF-1), resulting in an elevated tolerance for hypoxic conditions [96, 97]. However, hypoxia and the correspondingly increased HIF-1 activity may also cause an impairment in the expression of proteins required for an intact slit diaphragm, such as nephrin or podocin [98, 99]. Furthermore, HIF-1 can induce podocyte detachment due to cytoskeletal rearrangement, leading to proteinuria [99]. HIF-1 also causes TNF-dependent inflammation [100] and extracellular collagen accumulation, eventually causing fibrotic remodeling [101] (Fig. 2).

In the settings of traumatic, burn, and septic environments, hypoxia-, ischemia- and reperfusion-induced tissue damage contribute to AKI development. Reoxygenation and restoration of nutrient delivery to renal tissue initiates cellular responses and thus can lead to vascular malfunction and cell injury and death. These acute alterations also illicit chronic renal damage [102]. It is likely that acidosis, hypothermia, and coagulopathy (also known as the lethal triad in trauma) play an additional and, so far, underestimated role in podocyte function and resulting changes in the glomerular filter. Taken together, the role of podocytes in trauma, burn, and sepsis-related injuries is neither completely evaluated nor completely understood, and the impact of these acute diseases, particularly on the upstream region of the nephron, needs further research.

### Future molecular targets and therapeutic strategies

To prevent or ameliorate development of AKI upon exposure to trauma, sepsis, or burn injury, early symptomatic AKI treatment including resuscitation of the circulation by rational administration of fluids, vasoactive agents, and antibiotics, and limiting invasive ventilation is mandatory [103]. Concerning targeted therapies, several promising molecular targets have been suggested which, however, still await clinical transfer in the future: For example, in non-human primates, blockade of the central complement component C3 after trauma/hemorrhagic shock improved signs of TRAKI [104] and reduced the damage-driving TNK-1 [105], and similarly improved AKI in a septic shock model where C3-blockade reduced microthrombi within the glomeruli [106]. In *E. coli*-induced septic multiple organ failure in baboons, overall survival was improved by RA101295, a peptide inhibitor of complement C5 cleavage, associated with an improvement of histopathological signs of AKI. Of note, the C5 blockade resulted not only in the disappearance of tubular casts, but also in a significant reduction of intraglomerular microthrombi during sepsis [107]. A repurposed anti-malaria drug, artesunate, has previously been shown to improve trauma/hemorrhagic-shock-induced AKI in rats by attenuating the NF-KB pathway and iNOS expression in the kidneys without further spatial differentiation [108]. Similar results were achieved by inhibition of Bruton’s tyrosine kinase, which activates NF-KB and the NLRP3 inflammasome presumably contributing to AKI development [109]. However, future mapping of the temporospatial response within the kidneys after defined challenges will reveal further molecular targets within the glomerulus.

## Conclusion

Despite the evident enormous importance of AKI in the critically ill, there is a striking lack of knowledge on the molecular events in the glomerular filter with its intricate components during trauma, burn, and sepsis. Future studies in well-designed and clinically relevant AKI models may clarify the involved pathomechanisms in glomerular injury, and lead to further improvements in clinical care to preserve adequate kidney function and thus to improve the outcome.

## Abbreviations

AKI: Acute kidney injury
CLP: Cecal-ligation-and-puncture
COX-2: Cyclooxygenase-2
DAMPs: Damage-associated molecular patterns
FAO: Fatty acid oxidation
GBM: Glomerular basement membrane
GECs: Glomerular endothelial cells
GFR: Glomerular filtration rate
HO-1: Heme-oxygenase-1
HMGB-1: High-mobility-group-protein B1
HIF-1: Hypoxia-inducible factor-1
IL: Interleukin
IRI: Ischemia–reperfusion Injury
LPS: Lipopolysaccharide
MAMPs: Microbe-associated molecular patterns
MCP-1: Monocyte chemoattractant protein-1
NO: Nitric oxide
NFkB: Nuclear factor k-light-chain enhancer of activated B cells
NEAT1: Nuclear paraspeckle assembly transcript-1
PDGF: Platelet-derived growth factor
ROS: Reactive oxygen species
RVR: Renal vascular resistance
RAAS: Renin–angiotensin–aldosterone-system
SOD: Superoxide dismutase
TCC: Terminal complement complex
TNFR-1: TNF receptor-1
TEM: Transmission electron microscopy
TRAKI: Trauma-related acute kidney injury
TNF: Tumor necrosis factor
